# Left and Right Ventricular Volumes and Global Systolic Function in Isolated Left Bundle Branch Block: A Cardiac Magnetic Resonance Imaging Study

**DOI:** 10.1186/1532-429X-18-S1-P296

**Published:** 2016-01-27

**Authors:** Shadi Akhtari, Michael L Chuang, Sophie Berg, Kraig V Kissinger, Beth Goddu, Warren J Manning

**Affiliations:** grid.239395.70000000090118547Cardiology, Beth Israel Deaconess Medical Center, Boston, MA

## Background

Echocardiographic studies have shown a reduction in global left ventricular (LV) ejection fraction (LVEF) in patients with isolated left bundle branch block (LBBB). This is likely due to altered electromechanical activation and resultant loss of septal contribution to LV systolic function. We sought to characterize LV volumes and global systolic function in adult subjects with an isolated (not related to infarct or cardiomyopathy) LBBB and compared these data to matched adults without LBBB and were free of clinical cardiovascular disease, using Cardiac Magnetic Resonance (CMR) imaging.

## Methods

Subjects with isolated LBBB (n=18, 10F) were identified from the hospital echocardiographic database and invited to undergo CMR. Age-, sex-, and body-surface area (BSA)-matched controls (n=18) were identified from a large community-dwelling cohort who had undergone CMR, as part of a population study, on the same CMR scanner using the same SSFP sequence. Ten-mm thick contiguous slices in the LV short-axis orientation encompassing both ventricles were obtained. Right and left ventricular endocardial borders were manually segmented by a trained observer and volumes were determined in the standard fashion using a modified summation of disks method. Ejection fraction was computed as stroke volume divided by end-diastolic volume in each ventricle. Differences between the LBBB group and matched controls were assessed using the Wilcoxon paired signed rank test; a p < 0.05 was considered significant.

## Results

LBBB subjects ranged in age from 37 to 82 years. Left ventricular end-diastolic volume (EDV) and end-systolic volume (ESV) were larger in the LBBB group (p < 0.01), but LV stroke volume (p=0.13) and cardiac output (p=0.18) did not differ between LBBB subjects and controls. Subjects with isolated LBBB had significantly lower LVEF (56 ± 7%) than controls (68 ± 6%), p = 0.0002. Right ventricular volumes and ejection fraction did not differ between isolated LBBB and control groups.

## Conclusions

Using the reference standard of volumetric CMR, our study demonstrates that adults with an isolated LBBB have greater LVEDV and LVESV than age, sex and BSA-matched individuals without LBBB. While LV stroke volume and cardiac output did not differ between groups, LVEF was significantly lower in subjects with LBBB. Right ventricular volumes and ejection fraction did not differ between groups. These data are important for the clinical interpretation of left and right ventricular volumes and ejection fraction in LBBB patients referred for CMR.

**Table 1:** Baseline and biventricular characteristics of left-bundle branch block (LBBB) subjects and matched controls.

Age, years	61.3 ± 13.0	61.8 ± 12.0	0.20
BSA, m2	1.90 ± 0.24	1.89 ± 0.23	0.25
Heart Rate, /min	67 ± 11	65 ± 12	0.18
LVEDV, ml	145 ± 34	127 ± 28	0.009
LVESV, ml	65 ± 20	42 ± 14	0.0001
LV Stroke Vol, ml	81 ± 18	86 ± 16	0.13
LV EF, %	56 ± 7	68 ± 6	0.0002
LV CO, L/min	5.4 ± 1.4	5.3 ± 1.7	0.18
RVEDV, ml	122 ± 38	130 ± 40	0.08
RVESV, ml	46 ± 22	50 ± 21	0.17
RV Stroke Vol, ml	76 ± 21	80 ± 21	0.19
RV EF, %	64 ± 9	63 ± 6	0.21

